# Investigation of the Mechanical Strength of Artificial Metallic Mandibles with Lattice Structure for Mandibular Reconstruction

**DOI:** 10.3390/ma17143557

**Published:** 2024-07-18

**Authors:** Shinsuke Kawamata, Tadashi Kawai, Erika Yasuge, Isao Hoshi, Tadaharu Minamino, Shingo Kurosu, Hiroyuki Yamada

**Affiliations:** 1 Division of Oral and Maxillofacial Surgery, School of Dentistry, Iwate Medical University, 19-1, Uchimaru, Morioka 020-8505, Iwate, Japan; kawashi@iwate-med.ac.jp (S.K.); erikay@iwate-med.ac.jp (E.Y.); isaohosh@iwate-med.ac.jp (I.H.); yamadah@iwate-med.ac.jp (H.Y.); 2 Department of Elementary Material Process Technology, Iwate Industrial Research Institute, 2-4-25, Kitaiioka, Morioka 020-0857, Iwate, Japan; tada.minamino@gmail.com (T.M.); kurosu@pref.iwate.jp (S.K.)

**Keywords:** mandibular reconstruction, mechanical strength, metallic artificial bone, lattice structure

## Abstract

Mandibular reconstructive surgery is necessary for large bone defects. Although various reconstruction methods have been performed clinically, there is no mandibular reconstruction method that meets both sufficient strength criteria and the patient’s specific morphology. In this study, the material strength of the cylindrical lattice structures formed by electron-beam melting additive manufacturing using titanium alloy powder was investigated for mandibular reconstruction. The virtual strengths of 28 lattice structures were compared using numerical material tests with finite element method software. Subsequently, to compare the material properties of the selected structures from the preliminary tests, compression test, static bending test and fatigue test were conducted. The results showed that there were correlations with relative density and significant differences among the various structures when comparing internal stress with deformation, although there was a possibility of localized stress concentration and non-uniform stress distribution based on the lattice structure characteristics. These results suggest that the lattice structure of body diagonals with nodes and a cell size of 3.0 mm is a potential candidate for metallic artificial mandibles in mandibular reconstruction surgery.

## 1. Introduction

In oral and maxillofacial surgery, mandibular reconstruction is frequently performed in conjunction with surgery for tumors, inflammation, or fractures [1,2,3]. Free vascularized bone grafting is a major mandibular reconstruction method that is commonly performed in current clinical practice [4]. However, it has the disadvantage of being highly invasive [5]. Therefore, mandibular reconstruction using iliac particulate cancellous bone and marrow with a titanium mesh tray has been proposed as an alternative [6]. However, the risk of infection at the bone harvest site and material problems, such as fatigue-induced breakage of the titanium mesh tray, remain unsolved [7]. To solve these problems, titanium mesh trays have been fabricated by metal additive manufacturing methods with computer-aided design data using selective laser melting technology or electron-beam melting technology [8,9]. Custom-made mesh trays that account for the morphology of the mandible and occlusal force have been developed [10].

Artificial custom-made metal bones produced by additive manufacturing methods have been applied in the orthopedic field as a bone substitute [11,12]. However, the material conditions for application to the legs and arms that bear high loads remain unclear [13]. The lattice structure has a periodic cell structure that is expected to be highly rigid and lightweight, and it is also used in the aerospace field [14,15]. Unit cell structures, such as tetrahedrons, cubes, and gyroids, are used as porous artificial bone-substitute materials in medical experiments [16,17]. The periodic size of the unit cell structure affects porosity and mechanical strength [18]. However, there are few experimental reports on lattice-structured objects fabricated using metal additive manufacturing. Although another group reported the osteogenic and angiogenic abilities of a titanium alloy scaffold fabricated by metal additive manufacturing with bone marrow mesenchymal stem cells and vascular endothelial progenitor cells in rabbit femurs [19], this study focused on the adhesion and proliferation of cells on the scaffold and did not evaluate the mechanical strength of the scaffold; therefore, its effectiveness for large bone defects that are subject to load bearing was not indicated. Another study measured the mechanical strength of a cubic titanium lattice structure fabricated by metal additive manufacturing [20,21,22]. However, this study only investigated the cubic lattice structure and did not compare it with other structures. No studies have evaluated the morphology and function of multiple lattice structures to determine the optimal type and structure of metal materials with lattice structures that can be used as human bone substitutes.

To develop a new mandibular reconstruction method that does not rely on autologous bone grafting, this study aimed to investigate the conditions for a bone-substitute material with an optimal lattice structure fabricated by additive manufacturing with sufficient strength to withstand clinical applications.

## 2. Materials and Methods

### 2.1. Selection of Lattice Structures

This study analyzed 28 types of lattice structures included in the finite element analysis software (ANSYS Multiscale. Sim**^®^** 2021 R1, Cybernet Systems Co., Ltd., Tokyo, Japan), which are often used in engineering. A numerical material test was performed by predicting the virtual material property values of each lattice structure using ANSYS Multiscale. Sim**^®^** 2021 R1. The sample conditions of the simulation included a Young’s modulus of 120 MPa, which was the condition value of the material used for the specimen preparation, and a size of 30 × 30 × 30 mm. The correlation between the equivalent physical properties and the relative density of the structure was evaluated. The evaluation criteria were an elastic modulus similar to that of cortical bone (Young’s modulus 10–30 GPa) [23], a maximum bite force of 2000 N [24], and a fatigue strength that could withstand 6.7 million chewing cycles over 10 years [25]. Three lattice structures were selected based on the analysis for the preparation of specimens.

### 2.2. Preparation of Specimens

The specimens were designed using computer-assisted design software (Materialise Magics 27, Materialize, Leuven, Belgium) and prepared using an electron-beam manufacturing system (Arcam EBM AX2; General Electric Company, Boston, MA, USA). Ti-64 grade 23 D50 (AP&C, Boisbriand, QC, Canada). Titanium alloy powder with a particle size of 45–106 mm and an average particle size of 70 mm was used for this study. The standard recipe recommended by the equipment manufacturer was used to determine the molding conditions for the specimens.

### 2.3. Compression Test

Because standards for specimens with lattice structures have not yet been established, the Japanese JIS T 0309 [26], 0312 [27], or 0313 [28] standards are referred to as the standard. Multiple lattices were arranged side by side on the load-bearing cross-section so that the multiple lattices could exhibit mechanical properties as aggregates. Each specimen was prepared in a cylindrical shape with a diameter of 6 mm and height of 10 mm. Three types of unit cell sizes (3, 4, and 5 mm) were prepared for the three selected lattice structures. An Instron 8874 mechanical testing system (Instron, Norwood, MA, USA) was used for the compression tests. The cylindrical specimens were compressed in the long-axis direction with the melting surface set as the bottom surface. The test speed (V: mm/min) was set as follows, V = *ah* × 60 = 10^−3^ × 10 mm × 60 = 0.6 mm/min ≒ 1.0 mm/min, where *a* was a strain rate of 10^−3^/s and *h* was a specimen height of 10 mm. From the stress–strain curve obtained, the 0.2% offset load was evaluated under the condition of 0.05 mm for the offset displacement corresponding to a 0.2% displacement. Five specimens were prepared from each group and evaluated.

### 2.4. Static Bending Test

Each specimen was prepared in a cylindrical shape with a diameter of 6 mm and a height of 50 mm. Three types of unit cell sizes (3, 4, and 5 mm) were prepared for the three selected lattice structures. An Instron 8874 mechanical testing system was used in this study. A three-point bending test was also conducted. The distance between the loading and supporting rollers was set to 25 mm, and the diameters of the loading and supporting rollers were 10 and 4 mm, respectively. From the obtained load–displacement curve, the 0.2% offset load was evaluated under the condition of 0.05 mm for the offset displacement corresponding to a 0.2% displacement. Five specimens were prepared from each group and evaluated. 

### 2.5. Fatigue Test

Dumbbell-shaped specimens were used for the fatigue test. The specimen was gripped on both sides, and the test was performed on a centrally located cylindrical section measuring 6 mm in diameter and 40 mm in length. A unit cell of size 3 mm was prepared for the selected structure based on the compression and static bending test results. An Instron 8874 mechanical testing system was used in this study. The test conditions were as follows: the cyclic wave shape was sinusoidal, the load ratio was 0.1, the cyclic frequency was 10 Hz, and the number of cycles was the cumulative number of tests until the specimens were destroyed. When the number of cycles was 10^6^ or more, no breakage was defined. A preliminary tensile test was performed on the three samples, and the reference load value was determined. Ten specimens were prepared for evaluation. From the results, an S–N curve was generated; the vertical axis represented the maximum stress (S), and the horizontal axis represented the number of cycles (N). 

### 2.6. Statistical Analysis

All values are reported as mean ± standard deviation. Statistical analyses were performed for each experiment. One-way analysis of variance (ANOVA) was used to compare the mean value among groups. If the ANOVA was significant, Tukey’s multiple comparison analysis was used as a post hoc test.

## 3. Results

### 3.1. Selection of Lattice Structures by a Numerical Material Test

**Figure 1:**
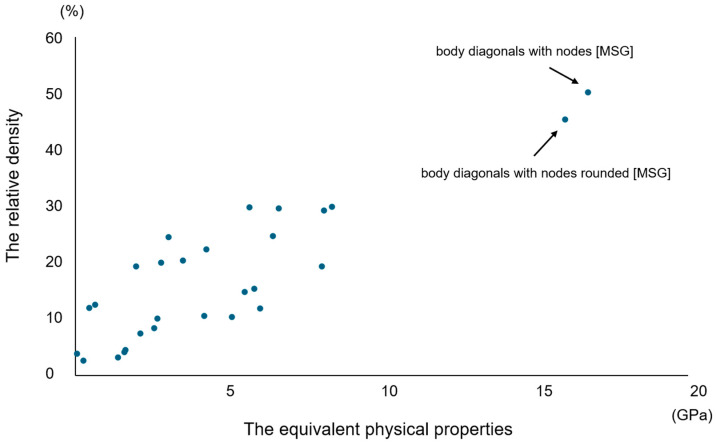
Equivalent physical properties and the relative density of each lattice structure. Structures that met the set criteria are body diagonals with nodes and body diagonals with rounded nodes, which are structures of the same type.

Table 1 lists the results of the numerical material tests for the 28 lattice structures. Figure 1 shows the positive correlation between the equivalent physical properties and relative density in each lattice structure. The structures that satisfied the set Young’s modulus condition (10–30 GPa) were body diagonals with nodes and body diagonals with rounded nodes, which were structures of the same type.

Diamond- and honeycomb-shaped lattice structures are rigid, and the stiffness depends on the length of the strut, cross-sectional area, and thickness of the shell. The relationship between the overall density and Young’s modulus was confirmed, and it was demonstrated that the lower the porosity, the higher the rigidity. The shape of the basic cells (triangular or square) indicates vulnerability in certain directions. Regarding the directionality of the lattice arrangement, a constant Young’s modulus was observed in all directions in the isotropic lattice arrangement; however, stiffness was observed in a specific direction in the anisotropic lattice arrangement.

**Figure 2:**
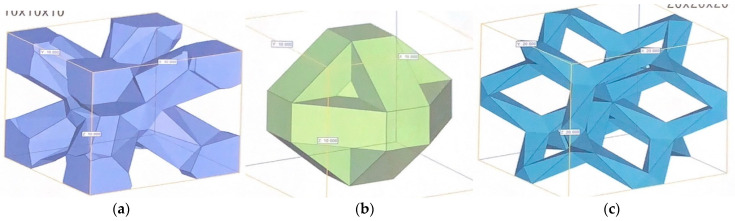
Images of lattice structures selected from the numerical material test. (**a**) BDN. A body-centered cubic lattice with nodes located at each vertex and the center of the cube. (**b**) G10. A two-dimensional honeycomb-like lattice, in which three graphene structures are bonded to one cell, forming a hexagonal network. (**c**) DM. Lattice structures based on dodecahedra.

To avoid bias in the structural strength, three lattices of body diagonals with nodes (BDN), G-structure 10 (G10), and dode—medium (DM) with different structural characteristics were selected as the specimen preparation conditions for the compression and static bending tests. Each lattice density was 0.50, 0.30, and 0.13 g/cm^3^, respectively, and their relative densities were 141.94, 84.54, and 35.63 g/cm^3^, respectively. In the bending fatigue test, BDN was selected as the specimen preparation condition. Figure 2 shows an image of each lattice structure. BDN is a body-centered cubic lattice with nodes located at each vertex and the center of the cube; because there is a fulcrum at the center of the diagonal, the structure constructed from its cells can be confirmed to have a constant isotropic Young’s modulus [29]. G10 is a two-dimensional honeycomb-like lattice in which three graphene structures are bonded to one cell, forming a hexagonal network [30]. DM is a lattice structure based on a dodecahedron, and although the shape of the basic cells makes it vulnerable in certain directions, it does have a certain degree of rigidity [31].

### 3.2. Compression Test

**Figure 3:**
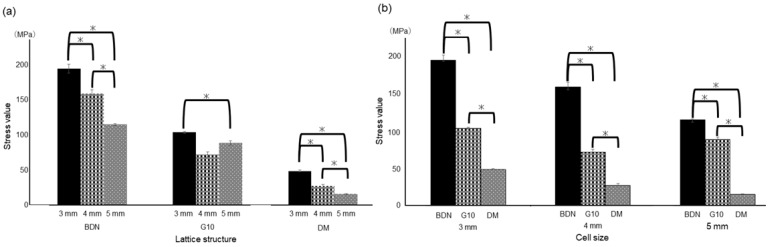
Result of the compression test. (**a**) Comparison of cell sizes for each lattice structure. For each of the three lattice structures, the cell size of 3 mm exhibited more stiffness than the cell sizes of 4 and 5 mm. (**b**) Comparison of the lattice structures for each cell size. BDN shows significant stiffness at each cell size compared to the others. Tukey’s multiple comparison analysis. * *p* < 0.05.

**Figure 4:**
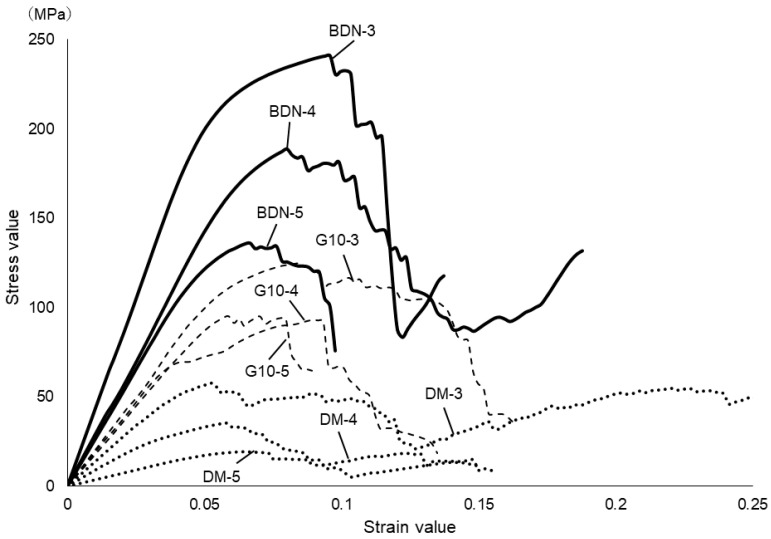
Stress–strain curve of the compression test. In BDN and G10, stress decreases rapidly after exceeding the maximum stress. In DM, the stress decreases slowly after the break. The straight line indicates BDN, the dashed line indicates G10, and the dotted line indicates DM. The number indicates the cell size.

Figure 3 shows the results of the compression test. The average 0.2% offset load values designed for the unit cell size of 3 mm for BDN, G10, and DM were 194.8 ± 6.4, 104 ± 1.3, and 48.7 ± 1.3 MPa, respectively. Those of 4 mm of BDN, G10, and DM were 159.0 ± 5.9, 72.2 ± 8.5, and 27.7 ± 2.4 MPa, respectively. Those of 5 mm of BDN, G10, and DM were 115.2 ± 1.1, 88.8 ± 3.2, and 16.1 ± 0.6 MPa, respectively. The cell size of 3 mm had a significantly higher offset value of 0.2% than the cell sizes of 4 and 5 mm. No significant difference was observed between the cell sizes of 4 mm and 5 mm in G10 (Figure 3a). The BDN showed significant stiffness for each cell size (Figure 3b). Figure 4 shows the stress–strain curves. In BDN and G10, the stress decreased rapidly after exceeding the maximum stress. However, the stress in the DM gradually decreased after breaking.

### 3.3. Static Bending Test

**Figure 5:**
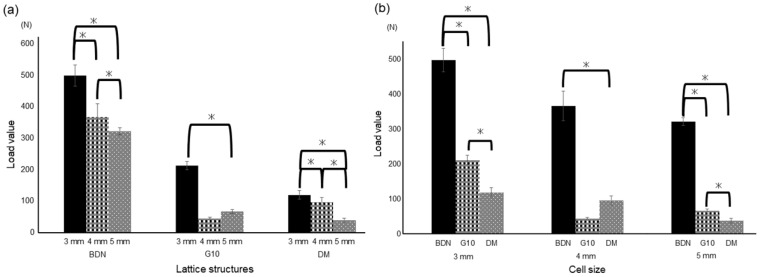
Results of the static bending test. (**a**) Comparison of cell sizes for each lattice structure. For each of the three lattice structures, the cell size of 3 mm exhibited more stiffness than the cell sizes of 4 and 5 mm. (**b**) Comparison of lattice structures for each cell size. BDN shows significant stiffness at each cell size compared to others. Tukey’s multiple comparison analysis. * *p* < 0.05.

**Figure 6:**
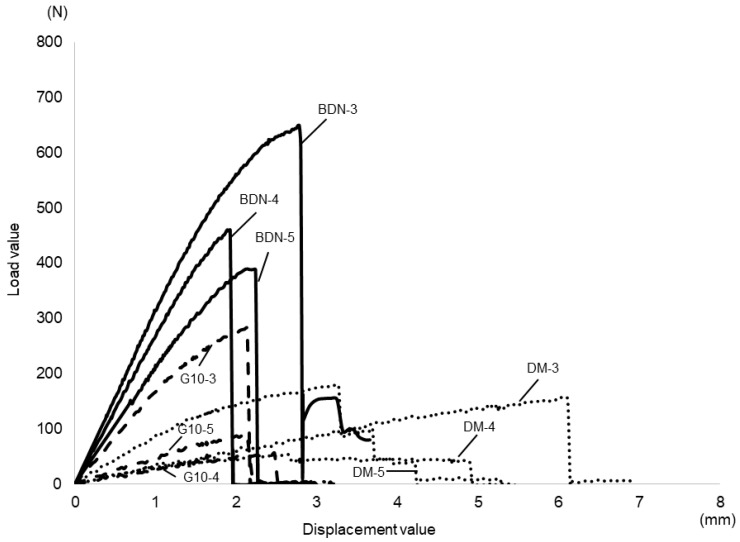
Load–displacement curve. Collapse occurs rapidly after the maximum stress in BDN. The straight line indicates BDN, the dashed line indicates G10, and the dotted line indicates DM. The numbers indicate the cell size.

Figure 5 shows the results of the static bending test. The average 0.2% offset load values designed for the unit cell size of 3 mm for BDN, G10, and DM were 498.0 ± 33.7, 212.0 ± 12.9, and 119.5 ± 13.3 N, respectively. Those of 4 mm of BDN, G10, and DM were 366.0 ± 42.4, 44.6 ± 4.1, and 96.6 ± 13.4 N, respectively. Those of 5 mm BDN, G10, and DM were 321.0 ± 11.1, 66.3 ± 6.1, and 38.6 ± 6.3 N, respectively. Among the three lattice structures, the cell size of 3 mm had a significantly higher offset value of 0.2% than the cell sizes of 4 and 5 mm. No significant difference was observed between the cell sizes of 4 mm and 5 mm in G10 (Figure 5a). The BDN showed significant stiffness for each cell size (Figure 5b). Figure 6 shows the load–displacement curves. Collapse occurred rapidly after the maximum stress in the BDN, but not in the others.

### 3.4. Fatigue Test

**Figure 7:**
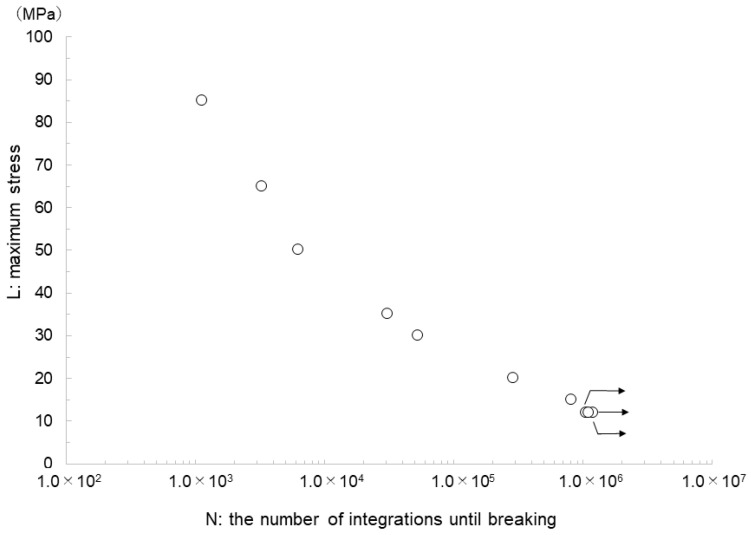
S–N curve from the fatigue test of BDN specimens. No breakage is observed at 12 MPa until 10^6^ cycles. Arrows indicate no breakage.

The reference load value was determined as 175.6 N based on the preliminary tensile test. Figure 7 shows the S–N curves. At maximum stresses of 85, 65, 50, 35, 30, 20, and 15 MPa, breakage was observed after 1125, 3288, 6252, 30,905, 52,969, 291,183, and 808,463 cycles, respectively. No breakage was observed at 12 MPa after 10^6^ cycles.

## 4. Discussion

In recent years, the application of biomaterials made by metal additive manufacturing has been considered in reconstructive surgery in various areas. Metal additive manufacturing is useful for improving the morphology and function of patient-specific defects, and the internal porosity provides stronger integration in the body and long-term durability [32]. The implant with the lattice structure has a more uniform pressure distribution at the interface between the implant and the bone than the non-lattice structure [33]. It has also been reported that adding a lattice structure to the root of dental implants improves initial stability [34]. Because the porosity of the lattice structure is important, a method has been reported to ensure that fabrication errors do not prevent the formation of porosity in lattice structures produced by laser additive manufacturing [35]. In clinical applications of artificial bones with a lattice structure, a clinical study on a prosthesis for a pelvic defect reported that it was useful and that no complications occurred [36]. In the oral and maxillofacial field, the application of artificial metal bone with a lattice structure customized to a patient’s alveolar defect has been reported [37]; however, there has been no evaluation of its mechanical strength. The artificial bone with a lattice structure in this present study not only reconstructed the defect but also demonstrated durability against mechanical stress around the mandible.

Various methods for mandibular reconstruction during oral surgery have been reported [38,39,40,41]. Reconstruction using titanium plates is simple, but it does not reconstruct the bone tissue and may lead to fatigue fracture [5]. Bone reconstruction using the ilium, fibula, and scapula involves the reconstruction of bone tissue; however, reconstruction of the ideal mandibular morphology is difficult. Mandibular reconstruction using a titanium mesh and iliac particulate cancellous bone and marrow can reconstruct the ideal mandibular morphology; however, there is a risk of titanium mesh fracture [42]. In this study, a titanium biomaterial with a lattice structure was designed for mandibular reconstruction that ideally reconstructs the mandibular shape, minimizes the use of grafted bone, and provides the appropriate strength. Furthermore, as the filling rate and pore size of the structure of cancellous bone is determined by the degree of internal stress and the anisotropy of the structure is determined by the direction of the internal stress, a lattice structure was proposed as an ideal structure that can prevent excessive stress concentration by continuously connecting structures with different local anisotropies while also ensuring the overall strength.

Material evaluation is based directly on the results of material testing; however, conducting a strength analysis of all lattice structure test specimens is expected to be extremely costly and time-consuming. Therefore, an evaluation was performed using finite analysis software. In the numerical material test, the apparent equivalent physical property values and relative density of the structure were compared, and a positive correlation was found between the equivalent physical property values and the Young’s modulus. The results indicated that the higher the relative density, the higher the Young’s modulus. Furthermore, because the type of lattice structure has a significant effect on mechanical properties, it may be possible to find an optimal structure by adjusting the topology, components, porosity, cell shape, and orientation of the lattice structure. In this study, because only two structures of the same type had a set Young’s modulus, one of them, BDN, was selected, and the other two, G10 and DM, which had different conditions, were selected to avoid bias in the structural strength.

The compression and static bending tests suggested that the smaller and more uniform the cells constituting the lattice, the higher the compressive strength, and that the compressive strength increased with the density of the structure. In BND and G10, the stress decreased rapidly after exceeding the maximum stress. They initially exhibited elastic deformation; however, when a certain stress was exceeded, plastic deformation might have occurred. However, stress did not decrease rapidly even after collapse occurred in the DM, probably owing to small breaks occurring intermittently rather than large breaks occurring until collapse occurred. The elastic modulus of each material exhibited different values depending on its structural characteristics. This strength was lower than that obtained in the numerical material test. This might be due to the weak bonding of the unit cells during specimen preparation [43].

The fatigue test for the BDN indicated a durability of 12 MPa. This value is approximately 4000 times the bite force of the molars in adults [44]. This suggests that a basic structure with a lattice structure of BDN and a cell size of 3.0 mm is effective for developing an artificial titanium mandible that can reconstruct the mandible shape of each patient using computer-aided design and manufacturing technology and the electron-beam additive manufacturing method that have been developed thus far, while also being strong enough to withstand biting forces.

This study investigated the lattice structure in the preparation of a metallic artificial mandible. In the future, it will be necessary to evaluate the reactivity of the body and mechanical strength of mandibular morphology. Artificial bones based on highly rigid and lightweight lattice structures are expected to be useful for mandibular reconstruction. However, as the reaction in the in vivo environment and long-term durability have not been confirmed, further investigation is required.

## 5. Conclusions

A basic lattice structure was investigated to develop a mandibular reconstruction method that would ideally reconstruct the mandibular shape, minimize the use of grafted bone, and achieve appropriate strength. When manufacturing titanium biomaterials with a lattice structure, BDN, which exhibits constant strength in an isotropic manner, has the optimal strength, and a structure with a cell size of 3.0 mm is optimal. Therefore, an artificial metal mandible with a lattice structure that meets these conditions could be a candidate for mandibular reconstruction. This method is expected to reduce the invasiveness to the patient by minimizing the amount of autologous bone harvested, and to be able to withstand biting forces and improve aesthetics. However, surface reactions in simulated body fluid environments and evaluation in in vivo environments have not yet been confirmed, so further research is needed before any clinical application.

## Figures and Tables

**Table 1:** Numerical material test of 28 lattice structures.

Structures	Equivalent Physical Properties (MPa)				Relative Density
	X Axis Direction	Y Axis Direction	Z Axis Direction	Average	
Body diagonals with rounded nodes	15,995	15,996	15,971	15,987	45.40%
Body diagonals with nodes	16,864	16,694	16,622	16,727	50.20%
Cross	5109	5109	5109	5109	10.40%
Cross-1	1390	1390	1390	1390	3.20%
Cross-2	1599	1598	1598	1598	4.20%
Cross-3	1635	1635	1635	1635	4.60%
Cross-X	348	23,333	434	8039	19.40%
Cross-X reinforced	36	699	45	260	2.70%
Diamond 20 percent relative density	2793	2793	2793	2793	20.00%
Diamond 30 percent relative density	6635	6635	6634	6635	29.60%
Dode—medium	630	630	630	630	12.60%
Dode—thick	3026	3027	3027	3027	24.60%
Dode—thin	47	47	47	47	3.90%
G-structure2	2149	3424	2150	2574	8.40%
G-structure3	553	212	552	439	12.00%
G-structure4	4206	4206	4206	4206	10.60%
G-structure6	6031	6031	6031	6031	11.90%
G-structure7	8052	443	8052	5515	14.80%
G-structure8	2519	1324	2520	2121	7.50%
G-structure9	3920	4003	4901	4275	22.40%
G-structure10	6619	7787	10,681	8362	29.90%
Octet truss relative density 30 percent	8120	8119	8100	8113	29.30%
Rhombi_Octa-Dense	5837	5837	5837	5837	15.40%
Rhombi_Octa-Light	2672	2672	2672	2672	10.10%
Rhombic dodecahedron relative density 20 percent	1964	1964	1975	1968	19.40%
Rhombic dodecahedron relative density 30 percent	5673	5673	5675	5674	29.80%
Trunc Octa-Dense	6513	6452	6348	6438	24.70%
Trunc Octa-Light	3590	3481	3458	3509	20.40%

## Data Availability

The raw data required to reproduce these results cannot be shared at this time, as the data also form part of an ongoing study.

## References

[1] Sjöström M., Danielsson D., Munck-Wikland E., Nyberg J., Sandström K., Thor A., Johansson H., Ceghafi P., Udd S.D., Emanuelsson J. (2022). Mandibular resection in patients with head and neck cancer: Acute and long-term complications after reconstruction. Acta Oto-Laryngol..

[2] Kasper R., Scheurer M., Pietzka S., Sakkas A., Schramm A., Wilde F., Ebeling M. (2023). MRONJ of the Mandible—From Decortication to a Complex Jaw Reconstruction Using a CAD/CAM-Guided Bilateral Scapula Flap. Medicina.

[3] Wongwaithongdee U., Inglam S., Chantarapanich N. (2023). Biomechanical evaluation of two internal fixation systems for the treatment of mandibular symphyseal fracture. Proc. Inst. Mech. Eng. Part H J. Eng. Med..

[4] Tarsitano A., Ceccariglia F., Bevini M., Breschi L., Felice P., Marchetti C. (2023). Prosthetically guided mandibular reconstruction using a fibula free flap: Three-dimensional Bologna plate, an alternative to the double-barrel technique. Int. J. Oral Maxillofac. Surg..

[5] Almansoori A.A., Choung H.W., Kim B., Park J.Y., Kim S.M., Lee J.H. (2020). Fracture of Standard Titanium Mandibular Reconstruction Plates and Preliminary Study of Three-Dimensional Printed Reconstruction Plates. J. Oral Maxillofac. Surg. Off. J. Am. Assoc. Oral Maxillofac. Surg..

[6] Zhao Z., Shen S., Li M., Shen G., Ding G., Yu H. (2023). Three-dimensional printed titanium mesh combined with iliac cancellous bone in the reconstruction of mandibular defects secondary to ameloblastoma resection. BMC Oral Health.

[7] Wang Q.S., Li S.J., Hou W.T., Wang S.G., Hao Y.L., Yang R., Misra R.D.K. (2020). Mechanistic understanding of compression-compression fatigue behavior of functionally graded Ti-6Al-4V mesh structure fabricated by electron beam melting. J. Mech. Behav. Biomed. Materials.

[8] Wang Z., Ummethala R., Singh N., Tang S., Suryanarayana C., Eckert J., Prashanth K.G. (2020). Selective Laser Melting of Aluminum and Its Alloys. Material.

[9] Zhang L.-C., Liu Y., Li S., Hao Y. (2018). Additive Manufacturing of Titanium Alloys by Electron Beam Melting: A Review. Adv. Eng. Mater..

[10] Hoshi I., Kawai T., Kurosu S., Minamino T., Onodera K., Miyamoto I., Yamada H. (2021). Custom-Made Titanium Mesh Tray for Mandibular Reconstruction Using an Electron Beam Melting System. Material.

[11] Wu N., Li S., Liu Y., Zhang A., Chen B., Han Q., Wang J. (2020). Novel exploration of 3D printed personalized total elbow arthroplasty to solve the severe bone defect after internal fixation failure of comminuted distal humerus fracture: A case report. Medicine.

[12] Meng M., Wang J., Huang H., Liu X., Zhang J., Li Z. (2023). 3D printing metal implants in orthopedic surgery: Methods, applications and future prospects. J. Orthop. Transl..

[13] Yang L., Yang L. (2015). 9-Safety of nanotechnology-enhanced orthopedic materials. Nanotechnology-Enhanced Orthopedic Materials.

[14] Maconachie T., Leary M., Tran P., Harris J., Liu Q., Lu G., Ruan D., Faruque O., Brandt M. (2022). The effect of topology on the quasi-static and dynamic behaviour of SLM AlSi10Mg lattice structures. Int. J. Adv. Manuf. Technol..

[15] Kim T., Zhao C.Y., Lu T.J., Hodson H.P. (2004). Convective heat dissipation with lattice-frame materials. Mech. Mater..

[16] Distefano F., Pasta S., Epasto G. (2023). Titanium Lattice Structures Produced via Additive Manufacturing for a Bone Scaffold: A Review. J. Funct. Biomater..

[17] Pałka K., Pokrowiecki R. (2018). Porous Titanium Implants: A Review. Adv. Eng. Mater..

[18] Sing S.L., Yeong W.Y., Wiria F.E. (2016). Selective laser melting of titanium alloy with 50 wt% tantalum: Microstructure and mechanical properties. J. Alloys Compd..

[19] Taniguchi N., Fujibayashi S., Takemoto M., Sasaki K., Otsuki B., Nakamura T., Matsushita T., Kokubo T., Matsuda S. (2016). Effect of pore size on bone ingrowth into porous titanium implants fabricated by additive manufacturing: An in vivo experiment. Mater. Sci. Eng. C Mater. Biol. Appl..

[20] Ushijima K., Akiyoshi T., Chen D.-H., Nakahara T., Cantwell W.J. (2013). Estimation of Bending Behaviour for Beams Composed of Three-Dimensional Micro-Lattice Cells (Part 1 Based on Numerical Analysis). Trans. Jpn. Soc. Mech. Eng. Part A.

[21] Ushijima K., Chen D.-H., Cantwell W.J., Seo M. (2010). Shear Deformation Response for Three-Dimensional Lattice Structures (1st Report, effects of geometry of overall lattice structure). Trans. Jpn. Soc. Mech. Eng. Part A.

[22] Ushijima K., Chen D.-H., Cantwell W.J., Seo M. (2011). Shear Deformation Response for Three-Dimensional Lattice Structures (2nd Report, Effects of Unit Cell Geometry in a Lattice Structure). Trans. Jpn. Soc. Mech. Eng. Part A.

[23] Hattori Y., Sato C., Watanabe M. (1996). Bite force distribution on dental arch during clenching. J. Jpn. Soc. Stomatognathic Funct..

[24] Satoh C. (1997). A Study of Bite Force Distribution on the Dental Arch in Normal Subjects. J. Jpn. Prosthodont. Soc..

[25] Dahlberg B. (1946). The masticatory habits; an analysis of the number of chews when consuming food. J. Dent. Res..

[26] (2009). Test Method for Fatigue Properties of Metallic Biomaterials.

[27] (2009). Testing Methods for Bending Properties of Metallic Osteosynthesis.

[28] (2009). Testing Methods for Compression Bending Properties of Metallic Osteosynthesis Devices.

[29] Ma X., Zhang N., Chang Y., Tian X. (2023). Analytical model of mechanical properties for a hierarchical lattice structure based on hierarchical body-centered cubic unit cell. Thin-Walled Struct..

[30] Tian W., Li W., Yu W., Liu X. (2017). A Review on Lattice Defects in Graphene: Types, Generation, Effects and Regulation. Micromachines.

[31] Xiao L., Song W., Wang C., Tang H., Fan Q., Liu N., Wang J. (2017). Mechanical properties of open-cell rhombic dodecahedron titanium alloy lattice structure manufactured using electron beam melting under dynamic loading. Int. J. Impact Eng..

[32] Davoodi E., Montazerian H., Mirhakimi A.S., Zhianmanesh M., Ibhadode O., Shahabad S.I., Esmaeilizadeh R., Sarikhani E., Toorandaz S., Sarabi S.A. (2022). Additively manufactured metallic biomaterials. Bioact. Mater..

[33] Distefano F., Epasto G., Guglielmino E., Amata A., Mineo R. (2023). Subsidence of a partially porous titanium lumbar cage produced by electron beam melting technology. J. Biomed. Mater. Res. Part B Appl. Biomater..

[34] Lee J., Li L., Song H.Y., Son M.J., Lee Y.M., Koo K.T. (2022). Impact of lattice versus solid structure of 3D-printed multiroot dental implants using Ti-6Al-4V: A preclinical pilot study. J. Periodontal Implant. Sci..

[35] Park J.W., Park H., Kim J.H., Kim H.M., Yoo C.H., Kang H.G. (2022). Fabrication of a lattice structure with periodic open pores through three-dimensional printing for bone ingrowth. Sci. Rep..

[36] Li Z., Luo Y., Lu M., Wang Y., Gong T., He X., Hu X., Long J., Zhou Y., Min L. (2024). Biomimetic design and clinical application of Ti-6Al-4V lattice hemipelvis prosthesis for pelvic reconstruction. J. Orthop. Surg. Res..

[37] Seiler M., Kämmerer P.W., Peetz M., Hartmann A. (2018). Customized lattice structure in reconstruction of three-dimensional alveolar defects. Int. J. Comput. Dent..

[38] Yagihara K., Okabe S., Ishii J., Amagasa T., Yamashiro M., Yamaguchi S., Yokoya S., Yamazaki T., Kinoshita Y. (2013). Mandibular reconstruction using a poly(L-lactide) mesh combined with autogenous particulate cancellous bone and marrow: A prospective clinical study. Int. J. Oral Maxillofac. Surg..

[39] Yang W.F., Choi W.S., Zhu W.Y., Su Y.X. (2020). “One-piece” patient-specific reconstruction plate for double-barrel fibula-based mandibular reconstruction. Int. J. Oral Maxillofac. Surg..

[40] Saijo H., Kanno Y., Mori Y., Suzuki S., Ohkubo K., Chikazu D., Yonehara Y., Chung U.I., Takato T. (2011). A novel method for designing and fabricating custom-made artificial bones. Int. J. Oral Maxillofac. Surg..

[41] Matsuo A., Chiba H., Takahashi H., Toyoda J., Abukawa H. (2010). Clinical application of a custom-made bioresorbable raw particulate hydroxyapatite/poly-L-lactide mesh tray for mandibular reconstruction. Odontology.

[42] Aytaç S., Ozbek S., Kahveci R., Ozgenel Y., Akin S., Ozcan M. (2005). Titanium mesh fracture in mandibular reconstruction. J. Craniofac. Surg..

[43] Smith M., Guan Z., Cantwell W.J. (2013). Finite element modelling of the compressive response of lattice structures manufactured using the selective laser melting technique. Int. J. Mech. Sci..

[44] Kobayashi R. (1990). Improvements in occlusion after orthognathic surgery. Jpn. J. Oral Maxillofac. Surg..

